# Association between bronchial asthma and TSLP gene polymorphism: a systematic review and meta-analysis

**DOI:** 10.1097/MS9.0000000000002107

**Published:** 2024-05-06

**Authors:** Abhigan B. Shrestha, Pashupati Pokharel, Harendra Singh, Sajina Shrestha, Shubham Shrestha, Yub Raj Sedhai

**Affiliations:** a Department of Internal Medicine, M Abdur Rahim Medical College, Dinajpur, Bangladesh; b Maharajgunj Medical Campus, Institute of Medicine, Tribhuvan University; c Department of Anesthesiology, Tribhuvan University Teaching Hospital, Kathmandu; d Department of Internal Medicine, KIST Medical College, Imadol; e Department of Internal Medicine, Patan Academy of Health Sciences, Lalitpur, Nepal; f Division of Pulmonary Disease and Critical Care Medicine, University of Kentucky, College of Medicine, Bowling Green, Kentucky

**Keywords:** asthma, meta-analysis, single nucleotide polymorphism, thymic stromal lymphopoietin, TSLP

## Abstract

**Aims:**

This study entails an association between bronchial asthma and common single nucleotide polymorphisms (SNPs) in thymic stromal lymphopoietin (TSLP) gene; rs2289278, rs3806933, rs2289276, and rs1837253.

**Methods:**

The databases of PubMed, Embase, Web of Science, and Google Scholar were searched for studies reporting TSLP polymorphisms and asthma from inception to January 2022. Hardy–Weinberg equilibriums (HWE) for each polymorphism of the control group were checked using the *χ* ^2^ test. The association was estimated by means of odds ratio (OR) with 95% CI in both dominant and recessive modes of inheritance, respectively.

**Results:**

Altogether, 11 studies with 3121 asthma cases and 3041 healthy controls were added. Results from six studies showed that the SNP rs2289278 had a protective role in asthma development (OR=0.65, 95% CI: 0.44–0.97, *P*=0.04). Pooling of four studies showed that the SNP rs3806933 had higher odds of developing asthma (OR=1.32, 95% CI:1.14–1.54, *P*<0.01). However, the SNP rs2289276 and rs1837253 showed no significant association. From the subgroup analysis, SNPs rs2289278 and rs1837253 were protective against the development of asthma in Asia. However, SNP rs2289276 showed a risk association in Asia and in adults.

**Conclusion:**

This meta-analysis shows that the SNP rs2289278 has a protective effect on the development of asthma; whereas rs3806933 has a risk of asthma. Additionally, this study adds genomic-based support to the recent FDA approval of tezepelumab, an anti-TSLP agent.

## Introduction

Highlights

- In the last two decades, the cytokine thymic stromal lymphopoietin (TSLP) has emerged as an important factor in the pathogenesis of asthma.
- Several genome-wide and single polymorphism studies have shown that multiple single nucleotide polymorphisms at the TSLP genomic locus are associated with increased asthma susceptibility.
- In this study of rs2289278, rs3806933, rs2289276 and rs1837253 gene polymorphism, the SNP rs2289278 somewhat had a protective effect, whereas rs3806933 had an increased risk of asthma.

Asthma is a chronic, reversible, obstructive airway inflammatory disease with variable expiratory airflow limitation presenting with wheeze, shortness of breath, chest tightness, and cough^1^. According to WHO, about 262 million people in the world are suffering from asthma^2^. The Global Burden of Disease 2018 report estimates that asthma accounts for ~420 000 deaths per year worldwide^3^. The interaction of genetic and environmental factors plays a critical role in the pathogenesis of asthma^4^.

There is growing evidence that genetic factors are involved in the development of asthma. In the last two decades, the cytokine thymic stromal lymphopoietin (TSLP) has emerged as an important factor in the pathogenesis of asthma^5–7^. TSLP, originally described as lymphocyte growth factor, is an epithelial cell-derived cytokine similar to IL-7^8^. The human TSLP gene is located on chromosome 5q22.1^9^. In general, TSLP is expressed mainly by the epithelium of the skin, lungs, and gastrointestinal tract. However, dendritic cells, mast cells, fibroblasts, and smooth muscles express TSLP under stimuli. It is implicated in the development and progression of allergic disorders like allergic rhinitis, atopic dermatitis, and bronchial asthma^10^.

Single nucleotide polymorphisms (SNPs) are the most common form of genetic variation in the human genome. Several genome-wide and single polymorphism studies have shown that multiple SNPs at the TSLP genomic locus are associated with increased asthma susceptibility in different ethnic backgrounds, sex, and age. Researches at a global scale have shown that the common SNPs in the TSLP promoter region associated with a higher risk of asthma are rs1837253, rs3806933, rs2289276, and rs2289278^11–21^.

Although TSLP is one of the crucial candidate genes for the development of allergic responses and the differentiation of T cells, formal statistical analyses between the SNPs of TSLP gene and the individual susceptibility to asthma have not been demonstrated. Current literature is contradictory about the association between different SNPs of the TSLP gene and asthma development in different countries. In an attempt to understand the genetic influence of TSLP on asthma, this meta-analysis was designed to understand the genetic influence of TSLP on asthma and estimate the association between a single nucleotide polymorphism and the risk of developing asthma.

## Methods

### Study protocol

The systematic review and meta-analysis were plotted according to the Preferred Reporting Items for Systematic Reviews and Meta-Analyses (PRISMA) guidelines. This systematic review and meta-analysis has been reported in line with PRISMA (Preferred Reporting Items for Systematic Reviews and Meta-Analyses) Guidelines^22^. The details of PRISMA checklist are presented in the Supplementary File (Appendix 1) (Supplemental Digital Content 1, http://links.lww.com/MS9/A464). This systematic review and meta-analysis has been reported in line with AMSTAR (Assessing the methodological quality of systematic reviews) Guidelines (Supplemental Digital Content 2, http://links.lww.com/MS9/A465)^23^. The predefined methodology with selection criteria was registered on PROSPERO.

### Search strategy

We searched the online databases of ‘PubMed’, ‘Embase’, ‘Web of Science’, and ‘Google Scholar’ for all related articles on TSLP polymorphism and asthma. The following keywords: ‘thymic stromal lymphopoietin’, ‘TSLP’, ‘polymorphism’, ‘variant’, ‘single nucleotide polymorphism’, ‘SNP’, ‘asthma’, and ‘asthmatic’ were used with suitable Boolean operators in between. Studies published from inception to January 2022 in any language were included. The references and citations of the originally retrieved articles, which were not captured by our database search, were searched manually. Authors of some studies were approached via Research Gate and email for reclamation of full text and missing data. The details of the search strategy are illustrated in the Supplementary File (Appendix 2) (Supplemental Digital Content 1, http://links.lww.com/MS9/A464).

### Selection criteria

Case–control studies published in any language from inception to January 2022 assessing the association between TSLP gene SNP polymorphisms (rs1837253, rs3806933, rs2289276, and rs2289278) and asthma were included. Case studies, reviews, conference abstracts, and studies without sufficient information were excluded. If the study subjects were reported in more than one publication, we selected the study with the largest sample size.

### Selection process

Although several SNPs in TSLP have been previously studied, only those most widely studied variants were analyzed and four SNPs (rs1837253, rs3806933, rs2289276, and rs2289278) were included in the analysis. The title and abstract of the articles identified through the electronic literature search were scanned. If the article could not be verified, the full text was further examined. The articles that met the following inclusion criteria were included in the analysis: case–control study; the association between TSLP (rs1837253, rs3806933, rs2289276, and rs2289278) and asthma; and sufficient data to perform the meta-analysis.

### Data extraction

Two reviewers (A.B.S. and S.S.) extracted the data independently. The following data were extracted: journal, first author, year of publication, country, genotyping methods, asthma diagnosis, average age at baseline, male percentage, distribution of genotypes for each polymorphism among cases and controls, and the odds ratio (OR) with their 95% CIs. Four polymorphisms were extracted, respectively, from the selected studies. The results of the items were compared and all of them reached consistency in the end.

### Quality assessment

Studies that matched the inclusion criteria were assessed for their quality appraisal using a tool proposed by the Joanna Briggs Institute for critical Appraisal of case–control studies. Two reviewers (A.B.S. and S.S.) independently evaluated the included studies and attributed a score to each ranging 0–10. Studies with a score >7 were reported as high quality, whereas scores 4–7 were reported as medium quality and a score <4 were reported as low quality, respectively. Any discrepancies between the two authors were solved by the fourth author (H.S.). The details of the quality assessment are included in the Supplementary File (Appendix 3) (Supplemental Digital Content 1, http://links.lww.com/MS9/A464).

### Statistical analysis

We examined the Hardy–Weinberg equilibrium (HWE) for each polymorphism of the control group by using the *χ* ^2^ test. The association between polymorphism and asthma was estimated employing ORs and corresponding 95% CIs comparing cases to controls. The dominant model (CT + TT vs CC), and recessive model (TT vs CC + CT) were used to estimate the statistical significance between TSLP polymorphisms and asthma association, respectively. The heterogeneity represents the total percentage of variation across the selected studies. We used the Chi-square-based *Q* statistic and *I* ^2^ statistics to evaluate the heterogeneity. The percentages of *I* ^2^ around 25, 50, and 75% indicate low, medium, and high heterogeneity, respectively. A *P*-value < 0.05 was representative of significant heterogeneity. When there was no significant heterogeneity (*I* ^2^ less than 75%), the fixed-effects model was used to calculate the pooled ORs; otherwise, the random-effects model was used.

In addition, we performed a sensitivity analysis to investigate the influence of the individual studies on the overall meta-analysis. Finally, the publication bias of the selected analysis was evaluated by funnel plot and Egger tests. We performed the analyses by using the STATA 16.0 software (StataCorp). A *P*-value < 0.05 was considered statistically significant and all statistical tests were two-sided.

## Results

### Study selection and characteristics

**Figure 1:**
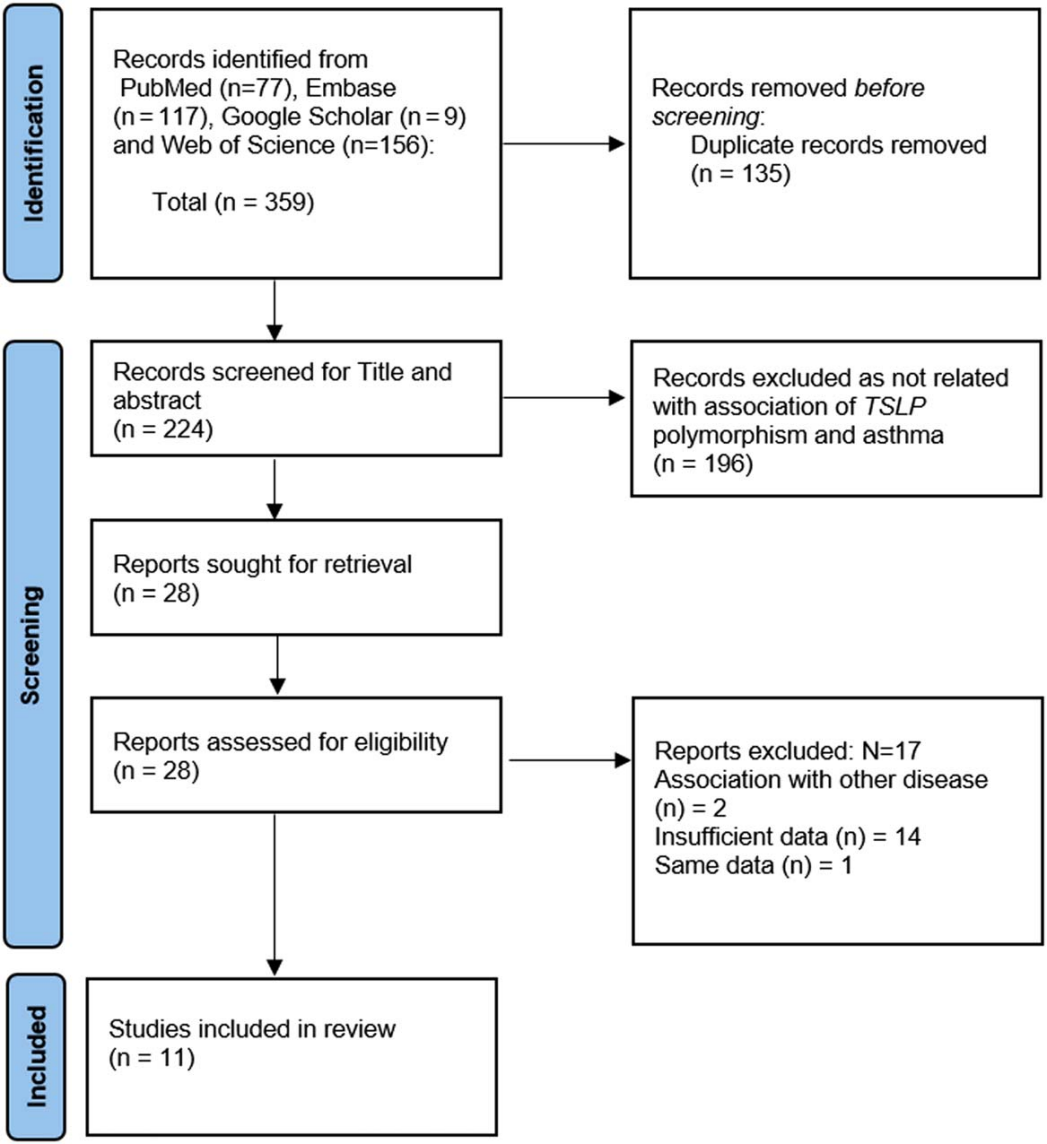
Preferred Reporting Items for Systematic Reviews and Meta-Analyses (PRISMA) flowchart detailing the study identification and selection process.

A total of 359 articles were identified by the keywords mentioned in the search strategy. After the removal of duplicate articles, 224 articles were screened from the title and abstract; of which 196 articles were excluded as they did not meet the inclusion criteria. The remaining 28 articles were screened for full-text, and 17 articles were removed for the reason that these articles had insufficient data for statistical analysis, or the subject had an association with other diseases, and full-texts were irretrievable. Finally, 11 studies were included into the meta-analysis. The PRISMA diagram depicting our study selection process is shown in Figure 1.

Altogether, 3121 asthma cases and 3041 healthy controls were included in the 11 studies within the time frame of 2011–2020. The sample size ranged from 40 to 2494. Studies were conducted on different continents: one from Africa, one from North America, one from Asia and Europe, and the remaining from Asia. The mean age of the cases was 25.53 years and of the controls was 25.72 years. Out of 11 studies, four had reported findings from children as well (below 18 years). The detailed characteristics of each study included in the analysis are presented in Table 1. Moreover, the Hardy–Weinberg equilibrium examination and genotype frequencies are depicted in Table 2.

**Table 1:** Characteristics of the included studies

		Case			Control			TSLP	TSLP	TSLP	TSLP
Author	Year	Number	Mean age	Male(*n*)	Number	Mean age	Male(*n*)	rs1837253	rs3806933	rs2289276	rs2289278
Moorehead *et al.* ^11^	2020	15	39	16	30	29.2	7	✓			
Mete *et al.* ^12^	2014	139	12.47	42	126	13.12	75		✓		
Elmaraghy *et al.* ^13^	2018	40	6.3	10	20	8.3	24			✓	✓
Ranjbar *et al.* ^14^	2020	126	35	66	300	36	32			✓	✓
Sun *et al.* ^15^	2019	123	39.06	52	100	28.34	50	✓			
Birben *et al.* ^16^	2014	506	9.2	78	157	10.4	309		✓		
Afzal *et al.* ^17^	2020	250	33.96	127	250	34.19	126	✓			
Harada *et al.* ^18^	2011	1280	30.55	730	1214	50.25	653		✓	✓	✓
Heo *et al.* ^19^	2018	20	27.1	10	20	27.1	13		✓	✓	✓
Wang *et al.* ^20^	2016	91	5.94	NA	284	5.5	NA				✓
Liu *et al.* ^21^	2012	531	42.3	257	540	40.6	227			✓	✓

Represents the SNP was studied.

NA, not available; SNP, single nucleotide polymorphism.

**Table 2:** Hardy–Weinberg equilibrium examination and genotype frequencies of each study.

					Case			Control			
Author	Year	Country	Case	Control	MM	MN	NN	MM	MN	NN	
rs1837253					CC	CT	TT	CC	CT	TT	p
Moorehead *et al.* ^11^	2020	Canada	15	30	7	7	1	12	11	7	0.18
Sun *et al.* ^15^	2019	China	123	100	23	69	30	11	41	48	0.617
Afzal *et al.* ^17^	2020	Pakistan	250	250	66	149	35	92	130	28	0.074
rs3806933 847C/T					CC	CT	TT	CC	CT	TT	p
Mete *et al.* ^12^	2014	Turkey	139	126	39	87	9	45	63	6	<0.01
Birben *et al.* ^16^	2014	NA	506	157	26	67	25	41	101	73	0.56
Harada *et al.* ^18^	2011	Japan	1280	1214	583	543	139	650	454	99	0.12
Heo *et al.* ^19^	2018	Korea	20	20	7	12	1	8	9	3	0.86
rs2289276 82C/T					CC	CT	TT	CC	CT	TT	p
Elmaraghy *et al.* ^13^	2018	Egypt	40	20	16	21	3	8	6	6	0.078
Ranjbar *et al.* ^14^	2020	Iranian	126	300	47	59	20	88	133	79	0.051
Harada *et al.* ^18^	2011	Japan	1280	1214	644	520	113	706	426	79	0.177
Heo *et al.* ^19^	2018	Korea	20	20	14	5	1	11	7	2	0.585
Liu *et al.* ^21^	2012	China	531	540	240	224	46	292	198	31	0.73
rs2289278 1560C/G					CC	CT	TT	CC	CT	TT	p
Elmaraghy *et al.* ^13^	2018	Egypt	40	20	38	2	0	20	0	0	1
Ranjbar *et al.* ^14^	2020	Iran	126	300	117	9	0	279	20	1	0.33
Harada *et al.* ^18^	2011	Japan	1280	1214	833	382	54	769	388	55	0.499
Heo *et al.* ^19^	2018	Korea	20	20	17	3	0	12	8	0	0.263
Wang *et al.* ^20^	2016	Taiwan	91	284	78	12	1	170	96	18	0.375
Liu *et al.* ^21^	2012	China	531	540	342	156	28	306	181	41	0.055

NA, not available.

### Study quality

The quality of the studies was assessed using the tool proposed by the Joanna Briggs Institute^24^. Out of 11 studies, 8 studies scored >7 and were of ‘high quality’, while 3 studies were of ‘medium quality’ with a score between 4 and 7. Finally, all 11 studies were included in the meta-analysis. Furthermore, the quality was checked using the HWE in the control group (Appendix 3). The study conducted by Mete *et al.* for variant rs3806933 did not satisfy the HWE, so it was excluded.

### Meta-analysis

#### Association between asthma and SNP rs2289278, rs3806933, rs2289276, and rs1837253 polymorphism

**Figure 2:**
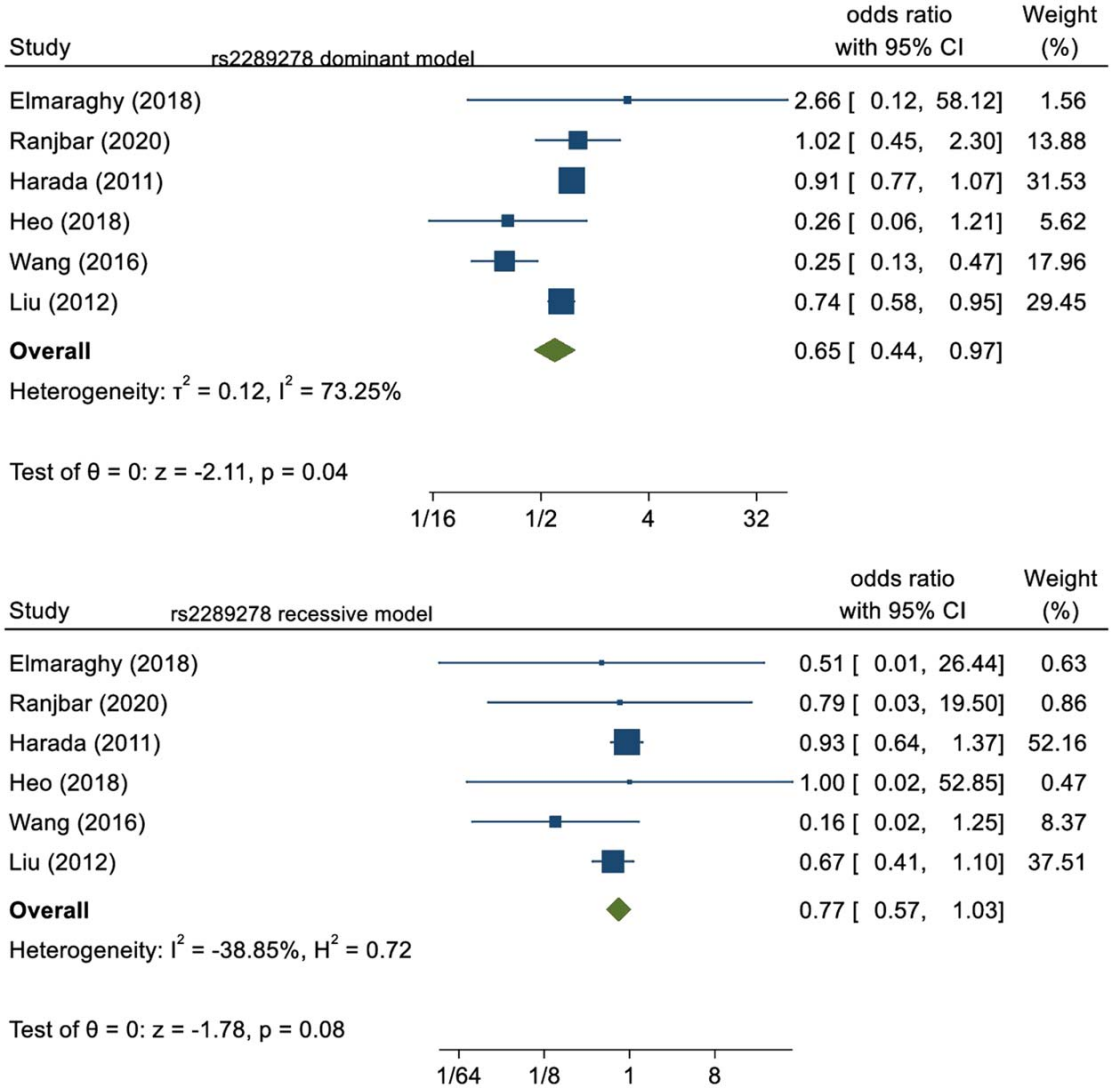
Forest plot of rs2289278 polymorphism in the dominant and recessive model.

Six studies analyzed the polymorphism of rs2289278 included in our study. They consisted of 2088 asthma cases and 2378 healthy controls. We pooled these eligible studies into the meta-analysis. Polymorphism of rs2289278 had a protective role for developing asthma in the dominant model (OR=0.65, 95% CI: 0.44–0.97, *P*=0.04) which was significant statistically; whereas in the recessive model (OR=0.77, 95% CI: 0.57–1.03, *P*=0.08) conclusion could not be brought statistically. This is illustrated in Figure 2.

**Figure 3:**
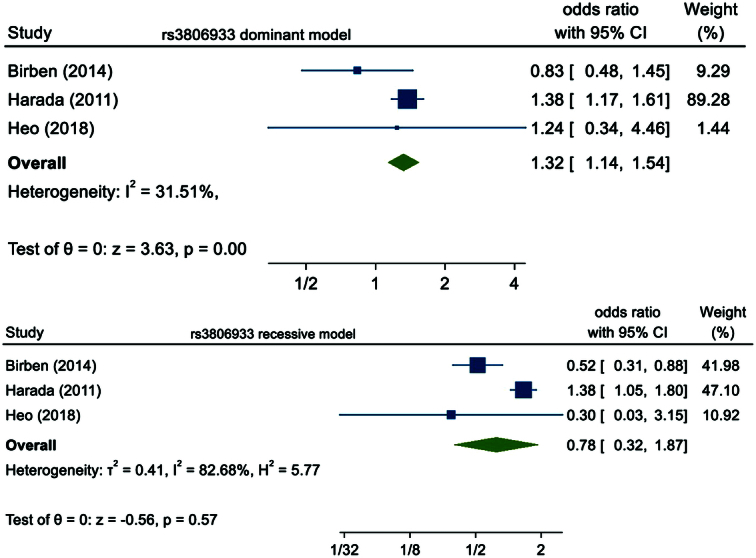
Forest plot of rs3806933 polymorphism in the dominant and recessive model.

Four studies analyzed the association of variant rs3806933 with the risk of asthma. A study conducted by Mete *et al.* was removed as it was not in HWE. The pooled three studies had altogether 1806 asthma cases and 1391 healthy controls. There was a significant risk associated between rs3806933 and asthma in dominant model (OR=1.32, 95% CI: 1.14–1.54, *P*< 0.01) but in the recessive model it was not statistically significant (OR=0.78, 95% CI: 0.32–1.87, *P*=0.57). This is illustrated in Figure 3.

**Figure 4:**
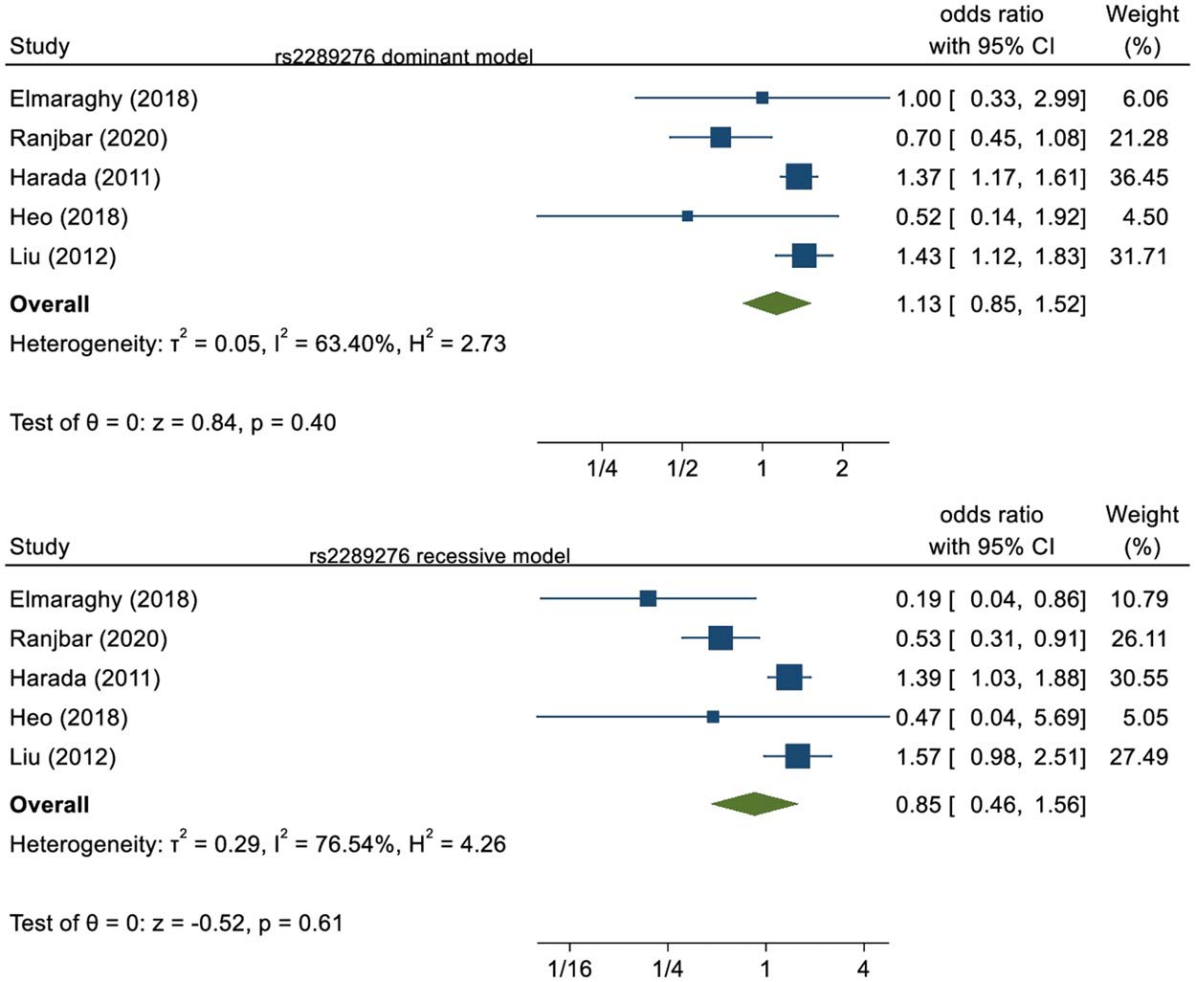
Forest plot of rs2289276 polymorphism in the dominant and recessive model.

Five studies analyzed the polymorphism of rs2289276 presented in our study. A total of 1997 cases and 2094 healthy controls were present. All these eligible studies were pooled for the association. No significant relation was seen in dominant model (OR=1.13, 95% CI: 0.85–1.52, *P*=0.40) as well as in recessive model (OR=0.85, 95% CI: 0.46–1.56, *P*=0.61). This is illustrated in Figure 4.

**Figure 5:**
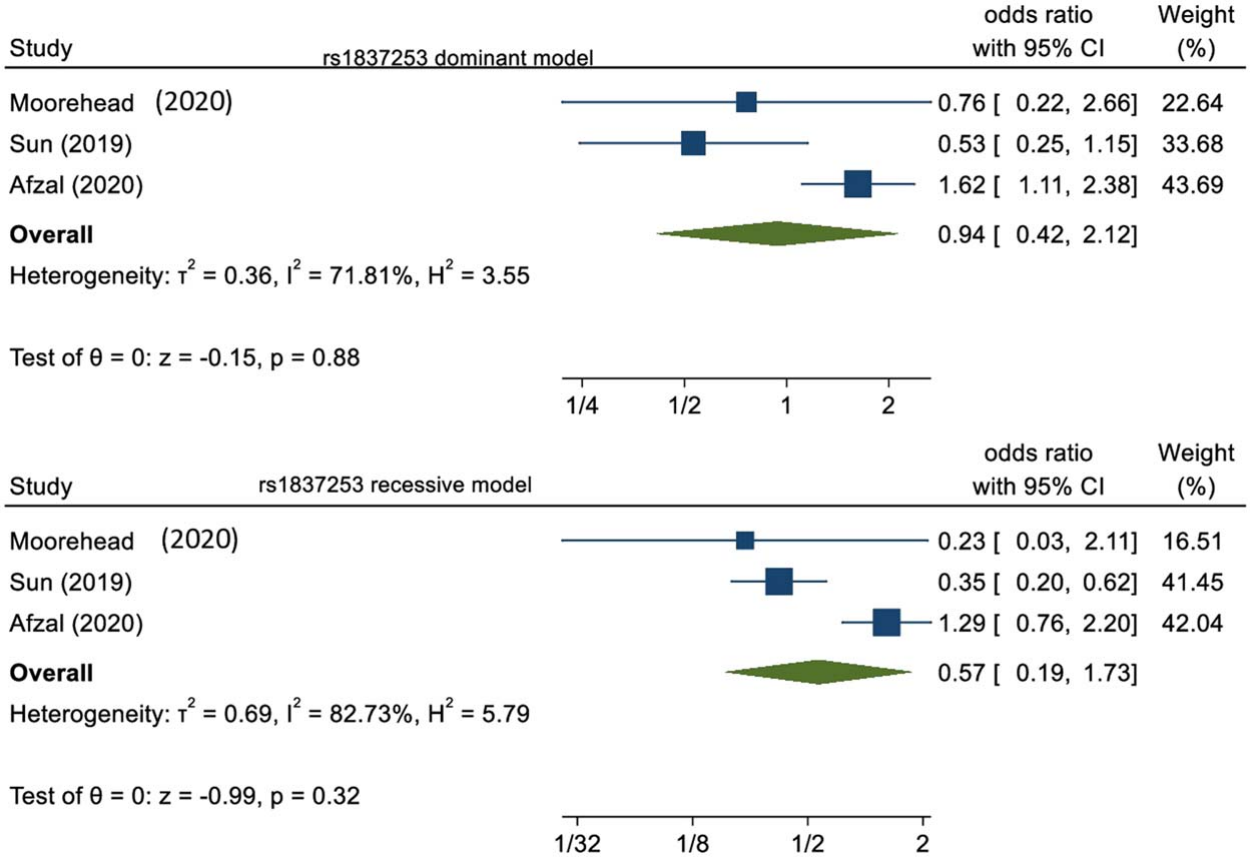
Forest plot of rs1837253 polymorphism in the dominant and recessive model.

Finally, three articles in our study analyzed the polymorphism of rs1837253. They had a total of 388 asthma cases and 380 healthy controls. After pooling these studies, protective nature was observed without statistically significance in dominant model (OR=0.94, 95% CI: 0.42–2.12, *P*=0.88) as well as in the recessive model (OR=0.57, 95% CI: 0.19–1.73, *P*=0.32). This is illustrated in Figure 5.

#### Sensitivity analysis

We evaluated the sensitivity of our analysis by omitting one study at a time. This had minimal impact on the overall result, thus confirming the stability and reliability of the pooled studies. Funnel plot and Egger’s regression test were used for assessing publication bias. Polymorphisms rs2289278, rs3806933, and rs1837253 and asthma risk studies did not suggest any publication bias, but rs2289276 variant and asthma study showed publication bias. The detail of the sensitivity analysis is presented in the Supplementary File (Appendix 4) (Supplemental Digital Content 1, http://links.lww.com/MS9/A464).

#### Subgroup analysis

Subgroup analysis was conducted for the four variants (rs2289278, rs3806933, rs2289276, rs1837253) for Age (children <18 years, adults >18 years) and continent. Supplementary File (Appendix 5) (Supplemental Digital Content 1, http://links.lww.com/MS9/A464).

For the variant rs2289278, protective role was seen in dominant model (OR= 0.582; 95% CI: 0.368–0.92; *P*<0.01) for Asia with statistical significance whereas no statistical association in recessive model (OR=0.768; 95% CI: 0.572–1.031; *P*=0.314). Similarly, no associations were discerned in adults and children in both models of inheritance.

For rs3806933, there was no significant association with the continent as well as for adults and children for both models.

For the variant rs2289276, risk association was observed with Asia continent (dominant model, OR=1.132; 95% CI: 0.823–1.55; *P*=0.013 and recessive model, OR=1.040; 95% CI: 0.598–1.810; *P*=0.009) and adults (Dominant model, OR=1.132; 95% CI: 0.823–1.55; *P*=0.013 and recessive model, OR=1.040; 95% CI: 0.598–1.810; *P*=0.009) in both model of inheritance which were verified statistically.

In subgroup analysis for rs1837253, both the dominant and recessive model showed decreased association with asthma in Asia (Dominant model, OR=0.98; 95% CI: 0.330–2.909; *P*=0.011, Recessive model, OR=0.678; 95% CI: 0.19–2.414; *P*=0.001) but no association with age category.

#### Publication bias

Funnel plot and Egger’s regression test were used for assessing publication bias. Polymorphisms rs2289278, rs3806933, and rs1837253 and asthma risk studies did not suggest any publication bias, but rs2289276 variant and asthma study showed publication bias. The funnel plot diagrams for publication bias analysis are shown in the Supplementary File (Appendix 6) (Supplemental Digital Content 1, http://links.lww.com/MS9/A464).

## Discussion

In this meta-analysis, 11 research papers were sought to measure the association of single nucleotide polymorphisms in thymic stromal lymphopoietin with asthma. A total of 3121 asthma cases and 3041 controls were evaluated for analysis and concluded that polymorphisms rs2289278 and rs3806933 in TSLP had a significant association with asthma.

The pathophysiology of asthma is based on the elevated level of cytokines like IL-4, IL-5, and IL-13 production from Th2 cells. Based on this research, targeting genetic polymorphisms that regulate the number of Th2 cells came to light. Besides, the classical Th2 cytokines, epithelial cell-derived cytokines like TSLP, IL-33, and IL-25, can initiate Th2 response at mucosal sites, and are secreted following tissue injury, epithelial stimulation, and pathogen pattern recognition or following allergen exposure^25–27^. TSLP expression can be regulated by inflammatory stimuli produced by both immune cells (i.e. innate and adaptive). The interaction between antigenic stimulation and TSLP produces airway inflammation with infiltration of eosinophils, airway remodeling, goblet cell metaplasia, and mucus overproduction. This connection may be a modifying factor for asthma phenotypes^28^.

**Figure 6:**
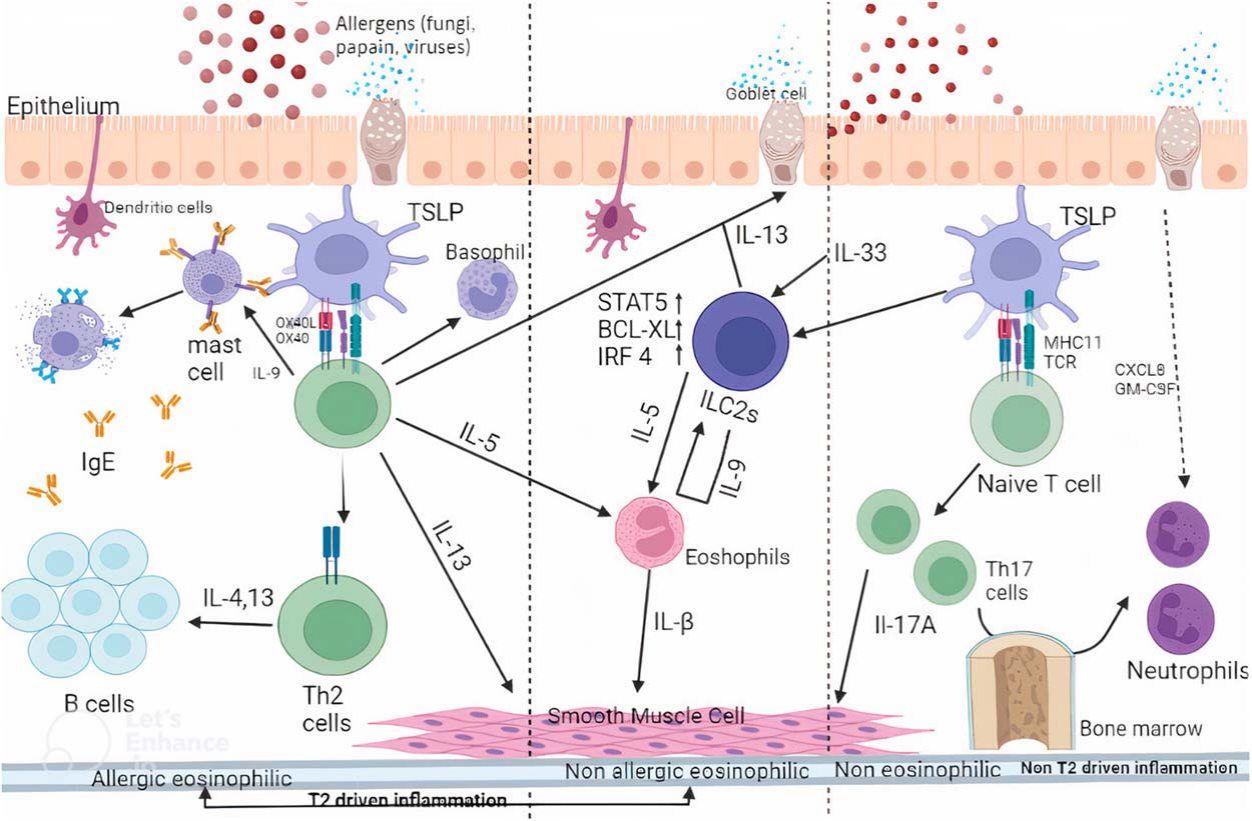
Thymic stromal lymphopoietin mechanism of action.

TSLP exerts its effect via TSLP-R composed of IL-7Rα chain paired with a TSLP-specific receptor component (TSLPR). Once the triple complex of TSLP/TSLPR/IL-7Rα forms, TSLP activates Janus kinase 1 (JAK1) and JAK2, thereby resulting in the phosphorylation of signal transducers and activators of primarily transcription 5A (STAT5A), STAT5B, and other STAT proteins, mitogen-activated protein kinases (MAPKs) and nuclear factor kappa B (NF-κB)^29,30^. The primary target of TSLP in humans is dendritic cells; therefore, it appears to influence mostly the sensitization stage of asthma. TSLP stimulated dendritic cells to increase OX40L expression and production of TH2 chemokines, such as CCL17 and CCL21, leading to the priming of CD4+TH2 cells and cytokine production by mast cells. This ultimately accelerates the survival and chemotaxis of eosinophils, cellular proliferation, and airway inflammation. The higher serum concentration of TSLP correlate with the severity of asthma, declining pulmonary functions^25,31,32^ and reduce corticosteroid sensitivity^33^. The sequential TSLP cascade is illustrated in Figure 6.

Two transcript variants are encoded by the TSLP gene: a long (lfTSLP) and short (sfTSLP) TSLP isoform. The two isoforms play opposite roles in the inflammatory process, the long isoform is associated with allergic inflammation, whereas the short isoform has anti-inflammatory or antimicrobial effects^34–36^. Moorehead *et al*.^11^ showed that despite both lfTSLP and sfTSLP being upregulated following stimulation with poly(I:C), lfTSLP was much more elevated feasibly due to its very low expression levels at a steady state.

One of the methods of discovering new genetic variants associated with complex diseases is a Genome-wide association study (GWAS)^37^. Nevertheless, asthma also accounts for a complex disease, which is usually caused by various genetic and environmental factors^38^. As such, GWAS study over a different population has shown novel variants; TSLP in association with asthma^5–7^. Such studies have not demonstrated all four variants (rs2289278, rs3806933, rs2289276, and rs1837253) in a single analysis. Even though with a smaller sample, accounting for the small effect of gene variants makes the data insignificant and inconclusive. Our meta-analysis overcomes this barrier by compiling to-date articles studying the four variants of TSLP.

With more than 200 polymorphisms associated with asthma, only a few of them have been reproduced^38^. Due to which genetic associations are challenging to identify. Harada *et al*.^36^ reported the association of rs3806933, rs2289276, and rs2289278 at first. In the study, an association was demonstrated between rs3806933 and rs2289276, but not with rs2289278. Whereas, Liu and Dong^39^ reported the association of variants rs2289276 and rs2289278 in the Chinese population, which showed the association of both variants in association with asthma. With each study, a concrete conclusion cannot be drawn out. Therefore, we attempted to analyze an extensive and up-to-date study concerning TSLP polymorphism and asthma.

In our paper, we present the protective nature of SNP rs2289278 (OR=0.65, 95% CI: 0.44–0.97, *P*=0.04). The SNP rs2289278 has a transcription-specific effect. This SNP is situated within an intron of the lfTSLP transcript but in the 5’untranslated area of the sfTSLP^18^. Among the four variants, the rs2289278 polymorphism mechanistic action was the least described. We hypothesize a pivotal role of sfTSLP in this variant for its protective role. A study by Harada *et al*.^18^ showed protective results for adults and children with asthma with rs2289278 but was not statistically proven, however, they showed a correlation with decreased lung function (FEV_1_/FVC). In another study by Wang *et al*.^20^, they mentioned the risk of asthma not only through lung function but also possibly through the allergic mechanism. The influence of the genetic association of the polymorphism on the role of the TSLP gene in asthma is not clear.

Similarly, we found a significant risk association of rs3806933 with asthma (OR=1.32, 95% CI: 1.14–1.54, *P*<0.01). The SNP rs3806933 (-847 C>T) in the promoter region of lfTSLP establishes a binding site for the transcription factor activating protein (AP-1) and the SNP enhances AP-1 binding to the regulatory element. In response to poly (I:C) stimulation, the functional variant increases the promoter-reporter activity of lfTSLP in normal bronchial epithelial cells. Therefore, in Th2 polarized immunity, the functional gene polymorphism of TSLP gene secretes higher TSLP production in response to viral respiratory infections^36^.

The SNP rs2289276 (-82C/T) had risk of asthma outcome (dominant model, OR=1.132; 95% CI: 0.823–1.55; *P*=0.013 and recessive model, OR=1.040; 95% CI: 0.598–1.810; *P*=0.009) in Asia. The risk could be explained by its mechanism as it alters the affinity of a transcription factor, AP-2 α, between the two alleles. The higher AP-2 α binding to the sequence containing -82C (on the protective allele) possibly suppresses its transcriptional activity through its repression effect^18^. Studies showed the association of this polymorphism protective for children^18^. In our analysis, we too had the same effect but could not be brought to conclusion statistically due to the paucity of studies conducted. This could also possibly explain the publication bias observed in this variant. In contrary, we found risk of asthma in case of adults (Dominant model, OR=1.132; 95% CI: 0.823–1.55; *P*=0.013 and recessive model, OR=1.040; 95% CI: 0.598–1.810; *P*=0.009) which has not yet been studied or explained.

The SNP rs1837253 had protective association with asthma in Asia (Dominant model, OR=0.98; 95% CI: 0.330–2.909; *P*=0.011, Recessive model, OR=0.678; 95% CI: 0.19–2.414; *P*=0.001). This SNP is situated 5.7 kb upstream of the TSLP transcription start site and is speculated to derange several possible transcription factor binding sites. There is also a possibility that this SNP can downregulate microbe-induced production of TSLP by inhibiting the regulatory elements by binding transcription factors^40^. In a study conducted by Moorehead *et al*.^11^, showed that individuals carrying the T allele had a lesser propensity for the occurrence of allergic inflammation than the major C allele, considering it as a ‘protective’. Differential responses are induced by SNP rs1837253 with a viral insult contributing to the inflammatory phenotype through a specific isoform^41^. Thus, it has a somewhat ‘protective’ effect on asthma risk.

In Asia, significant associations were seen in SNP rs2289278, rs2289276, and rs1837253. Despite asthma being less prevalent in Asia compared to other continents, the prevalence of wheeze is highest^42^. Trend of the rise of asthma seems to be inclining in Asia in which urbanization plays a major hand. Above all, environmental factors associated with asthma cannot be neglected, including viral infections, lifestyle, allergen exposure, or dietary factors^43,44^.

Considering an answer to the question whether therapy should be concerned for the raised TSLP level is that from our analysis, polymorphism plays a part in the TSLP alteration. This association makes TSLP a potential target for future therapeutics. Many studies have already anticipated anti-TSLP therapy to combat asthma or its uncontrolled cases^11,18,21^. It plays a vital role in the initiation and persistence of Th2-mediated airway remodeling and inflammation^45^. Its level is correlated with the disease severity in asthmatic patients^46^. In a study, TSLP expression was high despite the patients on high dose oral or inhaled corticosteroid therapy^47^, showing an association of TSLP with steroid resistance. In a mouse model study, blocking TSLP reduced airway remodeling and chronic allergic airway inflammation^45^. Similar result was illustrated in cynomolgus monkeys^48^. At the epithelial cell-DC interface, TSLP marks a major control for allergic inflammation, hence making it a promising target for the future immunotherapy of asthma^46^. Besides polymorphism, other factors drive the raised TSLP levels which should be focused on and study must be done. However, recently, owing these properties of TSLP, FDA has approved anti-TSLP tezepelumab as an add-on maintenance therapy to improve severe asthma to those uncontrolled on regular therapy^49^. Thus, TSLP has been growing as a field of interest to the researches around the globe and has raised many possibilities. This study adds the genetic perspective of TSLP and aids its positive possibility in the susceptibility of asthma, and thus the requirement of anti-TSLP agent.

Allergic diseases are greatly associated with gene-environment interactions. In this study, we found heterogeneity which was shown due to continent and age. Due to the paucity of data, we could not explore other sources of heterogeneity. According to Birben *et al*., the differences in studies might arise due to ethnic differences in the distribution of genotypes, the distribution of phenotypes, and the multifactorial causes of allergic disease. Another cause of genetic variation, which may define opposite effects on certain phenotypes of discrete cohorts, is the amount of exposure to an allergen in an individual^16^. Similarly, we had associations in our subgroup analysis with Asia, which might be explained due to the above reasons as well as the smaller size of the sample and the number of studies from different populations, ethnicities and continents. Likewise, other continents were not associated as not more than one article was retrieved. Moreover, even many data were not available to see the association with other models of inheritance like the additive model. Hence, the data must be interpreted cautiously.

Another hindrance to the interpretation is the lack of analysis of isoforms of TSLP gene by the studies included in the meta-analysis. Such a dichotomy affects the elucidation of population-based and gene-associated analysis. Most studies have concentrated on lfTSLP, which is induced and repressed by several toll-like receptors and glucocorticoids, but the role of sfTSLP has been less documented except for the part that it has antagonistic effects^34^. It has been shown in a study that long isoforms were more elevated than the short isoforms of TSLP rs1837253 on stimulation but did not find a significant association between asthmatic and nonasthmatic people^11^. Even more studies must be done to check the effects.

## Conclusion

This meta-analysis contributes the evidence that the polymorphisms rs2289278 and rs3806933 in TSLP were associated with asthma. The SNP rs2289278 somewhat had a protective effect, whereas rs3806933 had an increased risk of asthma. However, further studies with a larger sample size are needed to explain the association between other variations in TSLPs and asthma.

## Ethical approval

Not applicable.

## Patient consent

Not applicable.

## Source of funding

Not applicable.

## Author contribution

A.B.S.: conceptualization, methodology, software, validation, formal analysis, data curation, writing – original draft, and visualization; P.P.: methodology, writing – original draft, and writing – reviewing and editing; H.S.: writing – original draft and writing – reviewing and editing; S.S.: investigation, data curation, and writing – reviewing and editing; S.S.: writing – reviewing and editing; Y.R.S.: reviewing, editing, and supervision.

## Conflicts of interest disclosure

The abstract of this article was submitted and accepted at ATS 2024.

## Research registration unique identification number (UIN)

1. Name of the registry: PROSPERO.
2. Unique identifying number or registration ID: PROSPERO 2022 CRD42021261993.
3. Hyperlink to your specific registration (must be publicly accessible and will be checked): https://www.crd.york.ac.uk/prospero/display_record.php?ID=CRD42021261993

## Guarantor

Abhigan Babu Shrestha.

## Data availability statement

Data will be available on request to the corresponding author.

## Provenance and peer review

Not commissioned, externally peer-reviewed.

## Supplementary Material
